# The role of upfront primary tumor resection in asymptomatic patients with unresectable stage IV colorectal cancer: A systematic review and meta-analysis

**DOI:** 10.3389/fsurg.2022.1047373

**Published:** 2023-01-06

**Authors:** Zongyu Liang, Zhiyuan Liu, Chengzhi Huang, Xin Chen, Zhaojun Zhang, Meijuan Xiang, Weixian Hu, Junjiang Wang, Xingyu Feng, Xueqing Yao

**Affiliations:** ^1^ Department of Gastrointestinal Surgery, Second Department of General Surgery, The Sixth Affiliated Hospital, School of Medicine, South China University of Technology, Foshan, China; ^2^ Department of Gastrointestinal Surgery, Guangdong Provincial People’s Hospital, Guangdong Academy of Medical Sciences, Guangzhou, China; ^3^ Shantou University Medical College, Shantou, China; ^4^ Guangdong Provincial People’s Hospital Ganzhou Hospital (Ganzhou Municipal Hospital), Ganzhou, China; ^5^ School of Medicine, South China University of Technology, Guangzhou, China; ^6^ School of Biology and Biological Engineering, South China University of Technology, Guangzhou, China; ^7^ The Second School of Clinical Medicine, Southern Medical University, Guangzhou, China

**Keywords:** primary tumor resection, colorectal cancer, asymptomatic, unresectable, overall survival

## Abstract

**Background:**

Controversy exists over the role of upfront primary tumor resection (PTR) in asymptomatic patients with unresectable stage IV colorectal cancer (CRC). The purpose of this study was to evaluate the effect of upfront PTR on survival outcomes and adverse outcomes.

**Methods:**

Searches were conducted on PubMed, EMBASE, Web of Science, and Cochrane Library from inception to August 2021. Studies comparing survival outcomes with or without adverse outcomes between PTR and non-PTR treatments were included. Review Manager 5.3 was applied for meta-analyses with a random-effects model whenever possible.

**Results:**

Overall, 20 studies with 3,088 patients were finally included in this systematic review. Compared with non-PTR, upfront PTR was associated with better 3-year (HR: 0.69, 95% CI, 0.57–0.83, *P* = 0.0001) and 5-year overall survival (OS) (HR: 0.77, 95% CI, 0.62–0.95, *P* = 0.01), while subgroup analysis indicated that there was no significant difference between upfront PTR and upfront chemotherapy (CT) group. In addition, grade 3 or higher adverse effects due to CT were more frequent in the PTR group with marginal significance (OR: 1.74, 95% CI, 0.99–3.06, *P* = 0.05), and other adverse outcomes were comparable.

**Conclusions:**

PTR might be related to improved OS for asymptomatic patients with unresectable stage IV CRC, whereas receiving upfront CT is a rational alternative without detrimental influence on survival or adverse outcomes compared with upfront PTR.

**Systematic Review Registration:**

https://www.crd.york.ac.uk/prospero/display_record.php?RecordID=272675

## Introduction

Colorectal cancer (CRC) is the third most commonly diagnosed malignant tumor and the second leading cause of cancer death worldwide (1). Approximately 20% of newly diagnosed CRCs have reached stage IV with distant metastases, most of which are unresectable (2). It is practically inevitable for patients with primary tumor-related symptoms to receive primary tumor resection (PTR) or other interventional treatments such as diverting colostomy, colonic stenting, and decompression tube in case of obstruction, perforation, or hemorrhage. For asymptomatic patients, systemic therapy as the upfront treatment has shown acceptable tolerance, with only 16% of patients require intervention caused by subsequent primary tumor-related complications (3). Although it has been of great interest and actively debated in recent years, the role of upfront PTR remains controversial. On one hand, potential risks of emergency cases due to primary tumors can be prevented once it has been resected. On the other hand, the occurrence of postoperative adverse events or complications might delay the initiation of systemic therapy, and consequently impact patients' survival outcomes (4–6). Hence, the pros and cons of PTR need to be cautiously assessed in order to help guidelines development especially for a defined patient population with this specific condition.

Previously published studies reported conflicting results on this issue, with the result that the conclusions of relevant systematic reviews and meta-analyses have updated and changed from endorsement (7, 8) to reservation (9). All previously published meta-analyses included only retrospective studies due to the lack of prospective research before then. Recently, two randomized controlled trials (RCTs) from Korea (10) and Japan (11) were published respectively in 2020 and 2021, and both suggesting that PTR followed by chemotherapy (CT) showed no or no significant survival benefit over upfront CT.

With the emergence of more and more high-level evidence, an updated systematic review and meta-analysis is needed in time for current practice. This study aims to evaluate the effect of upfront PTR on survival outcomes and adverse outcomes, and so determine whether PTR should be performed in case of asymptomatic patients with unresectable stage IV CRC at the time of diagnosis.

## Methods

### Study selection

This systematic review and meta-analysis was performed following the Preferred Reporting Items for Systematic Reviews and Meta-Analyses (PRISMA) guidelines (12). This study has been registered at the International Prospective Register of Systematic Reviews (CRD42021272675).

Comprehensive literature searches were identified in the databases of PubMed, EMBASE, Web of Science, and Cochrane Library from inception to August 2021. The full search strategy is available in Supplementary Tables S1–4. Two reviewers (Z. Liu and Z. Liang) independently screened the titles and abstracts of identified studies. After duplicate studies were excluded, the full texts of screened papers were reviewed for further inspection. In addition, we attempted to contact the authors of reports without sufficient data.

Reporting meeting the criteria were considered eligible: (a) patients with stage IV CRC; (b) patients were asymptomatic or relatively symptom-free; (c) patients diagnosed and evaluated as incurable or unable to achieve curative surgery; (d) studies reporting at least survival outcomes. The exclusion criteria were as followed: (a) studies not eligible in accordance with the inclusion criteria; (b) studies without sufficient data; (c) reviews, letters, comments, conference abstracts, or reports only with a protocol.

### Data collection process

Variables of characteristics included year, country, study design, enrollment interval, number of participants, age, gender, and other clinicopathological features.

The primary outcomes were long-term survival outcomes, including overall survival (OS) and progression-free survival (PFS) of the PTR group and non-PTR group. The secondary outcomes consisted of postoperative complications and adverse events of both groups.

Data were independently extracted by two reviewers (Z. Liang and Z. Liu). Discrepancies were tackled through discussion with a third reviewer (XF).

### Quality assessment

The modified Jadad quality scale (13) ranging from 0 to 7 points was used for bias assessment of RCTs and the Newcastle-Ottawa scale (NOS) (14) ranging from 0 to 9 points was for non-RCTs in this systematic review, with higher scores indicating better quality. Studies scoring greater than or equal to 4 points of the modified Jadad scale or 5 points of the NOS were considered high quality.

Furthermore, funnel plots were also used to evaluate bias including selection bias, publication bias, true heterogeneity, poor methodological quality of smaller studies, and so on. Bias can be visually measured by whether the funnel diagram is symmetrically distributed, but it is subjective, and the interpretation results vary for different reviewers.

Quality assessment was rated by two review authors (Z. Liang and Z. Liu). In case of disagreements, a third author (XF) was asked to participate in discussion until a consensus is reached.

### Statistical analysis

Review Manager (Revman) 5.3 (https://community.cochrane.org/help/tools-and-software/revman) was applied for meta-analyses with a random-effects model whenever possible. Survival outcomes were presented as hazard ratios (HRs) with 95% confidence intervals (CIs). If HRs of included studies were not reported directly, an estimated HR was derived from Kaplan-Meier curves based on the method raised by Tierney et. al (15). In addition, continuous variables were analyzed by weighted mean differences (WMDs) with 95% CI, and odds ratios (ORs) with 95% CIs were used to assess dichotomous variables. All results compared were considered statistically significant at a two-sided *P* < 0.05.

The heterogeneity was evaluated by the Cochrane Q test and Higgins *I*^2^ test. If the heterogeneity was considered high (*P* < 0.1 or *I*^2^  > 50%), then a subgroup analysis or sensitivity analysis would be conducted.

## Results

### Study selected

**Figure 1:**
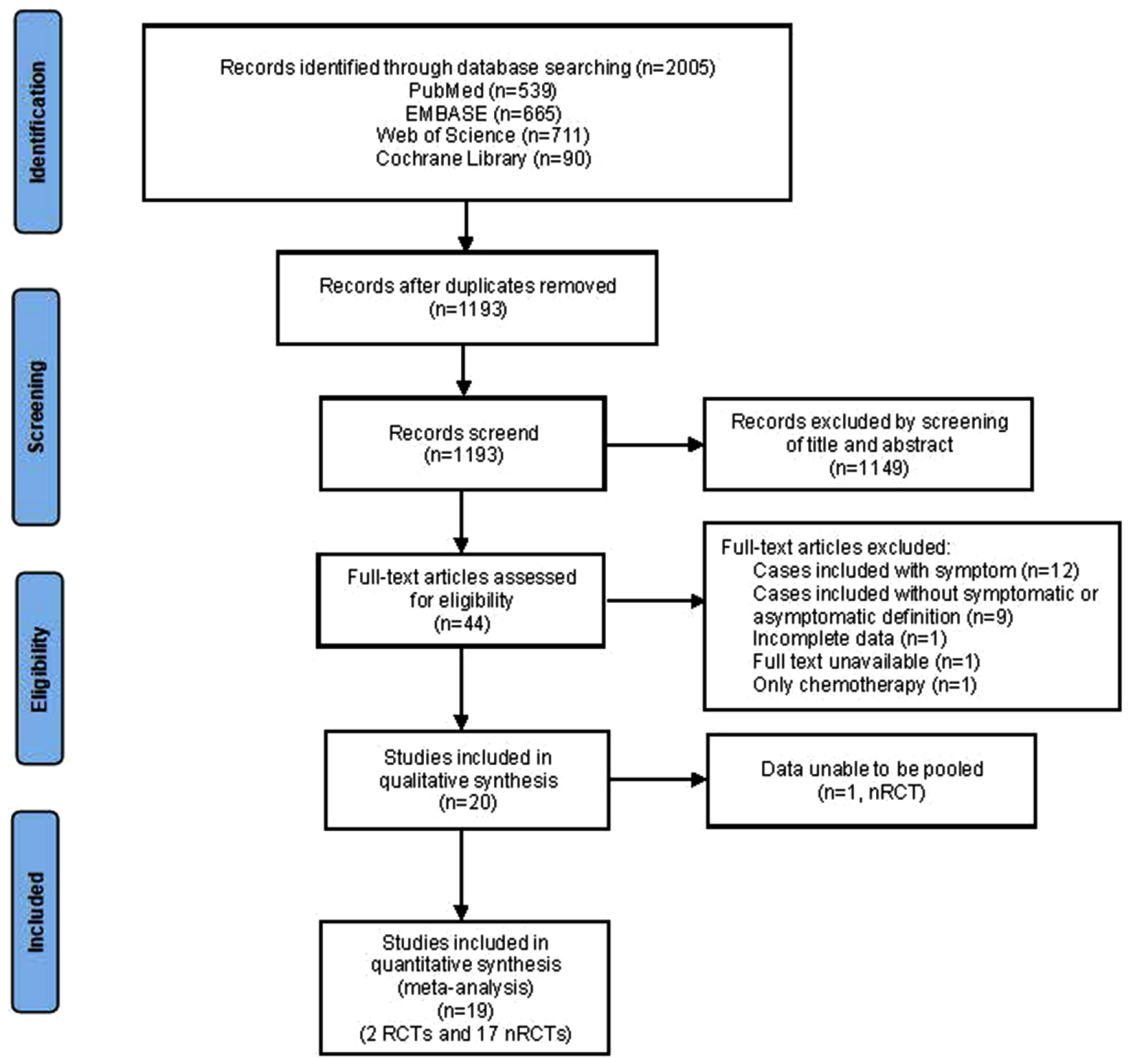
PRISMA selection flow diagram.

In total, 2005 studies were identified through databases, and 1,193 were left out after removing the duplicates. Of these, 44 studies requiring full-text assessment for eligibility remained. After further reviewing, a total of 20 published articles including 2 RCTs and 18 non-RCTs met the criteria. The flow chart demonstrating the details of the selection process was presented in Figure 1.

### Quality assessment

The quality of the included non-RCTs studies ranged from four to eight according to the NOS, and the median score was six points. Both two RCTs scored five points on the modified Jadad scale. Funnel plots were depicted for 3-year OS and 5-year OS with roughly symmetric distribution, suggesting that nearly barren of publication bias existed, even though there might be certain bias and heterogeneity.

Quality assessment of included studies was presented in Supplementary Table S5 and Figure S1.

### Study characteristics

In total, 20 studies (10, 11, 16–33) including 3,088 patients were finally included in this study. The baseline characteristics of each study were collected, listing in Table 1. These articles were published ranging from 1999 to 2021. The only two published RCTs (10, 11) were released in 2020 and 2021 respectively. In summary, 1,667 (54%) patients with asymptomatic and unresectable stage IV CRCs received PTR. The median follow-up period reported by included studies ranged from 7 to 46.5 months. The median age was concentrated around 60–65 years old. The proportion of males suffering from the disease was higher than that of females. Patients in studies of Benoist et al. and Galizia et al. were reported only with live metastases. Additionally, we summarized the clinicopathological characteristics of all the included studies. Notably, the clinicopathological features of eight studies (17, 18, 20, 21, 26, 28, 30–32) were not matched, which might contribute to bias or heterogeneity.

**Table 1:** Baseline characteristics of studies included.

Study	Design	Number of participants	Number of resections (%)	Median age (range), years old	Male/Female	Characteristics matched	OS benefits significantly from PTR
				PTR	non-PTR			PTR	non-PTR
Scoggins, USA, 1999	Prospective	89	66 (74%)	64 (37–89)	61 (44–93)	NA	NA	Yes	No
Ruo, USA, 2003	Prospective	230	127 (55%)	NA	NA	NA	NA	No	Yes
Michel, France, 2004	Prospective	54	31 (57%)	59.8^b^	58.9^b^	17/14	16/7	No	No
Benoist, France, 2005	Prospective	59	32 (54%)	60^b^	61^b^	19/13	18/9	Yes	No
Galizia, Italy, 2008	Prospective	65	42 (65%)	62 (28–84)^b^	59 (28–82)^b^	28/14	15/8	Yes	Yes
Seo, Korea, 2010	Prospective	227	144 (63%)	58^b^	56^b^	55/29	12/5	No	No
Boselli, Italy, 2013	Prospective	48	17 (35%)	70 (54–84)^b^	73 (60–87)^b^	NA	NA	Yes	No
Cetin, Turkey, 2013	Prospective	99	53 (54%)	55 (28–73)	52 (23–74)	29/24	27/19	Yes	Yes
Matsuda, Japan, 2013	Prospective	46	40 (87%)	NA	NA	NA	NA	NA	Yes
Matsumoto, Japan, 2014	Prospective	88	41 (47%)	67 (59–72)^a^	62 (57–68)^a^	25/16	33/14	Yes	No
Watanabe, Japan, 2014	Prospective	158	46 (29%)	63 (41–81)	60 (27–80)	25/31	71/41	No	No
Yun, Japan, 2014	Prospective	416	218 (52%)	58 (23–87)	59 (25–77)	141/77	130/68	No	No
Samalavicius, Lithuania, 2016	Prospective	226	113 (50%)	66 (37–91)	67/53	37/26	No	Yes
Zhang, China, 2017	Prospective	263	125 (48%)	NA	NA	NA	NA	NA
Liang, China, 2018	Prospective	411	278 (68%)	57.2 ± 11.9^b^	56.0 ± 10.1^b^	145/133	65/68	No	No
Ergun, Turkey, 2020	Prospective	147	56 (38%)	58 (26–80)	56 (25–80)	36/20	61/30	No	Yes
Park, Korea, 2020	RCT	48	26 (54%)	62.3 (40–81)	58.8 (32–74)	21/5	12/10	Yes	Yes
Urvay, Turkey, 2020	Prospective	215	76 (35%)	59 (22–85)	62 (27–86)	85/54	51/25	No	Yes
Doah, Korea, 2021	Prospective	146	98 (67%)	69 (58–77)^a^	66.5 (62–75)^a^	49/49	29/19	Yes	No
Kanemitsu, Japan, 2021	RCT	165	81 (49%)	65 (59–69)^a^	65 (59–71)^a^	45/36	45/39	Yes	No

^a^ Presented with median (interquartile range).

^b^ Presented with mean or mean (standard deviation).

### Primary outcomes

**Figure 2:**
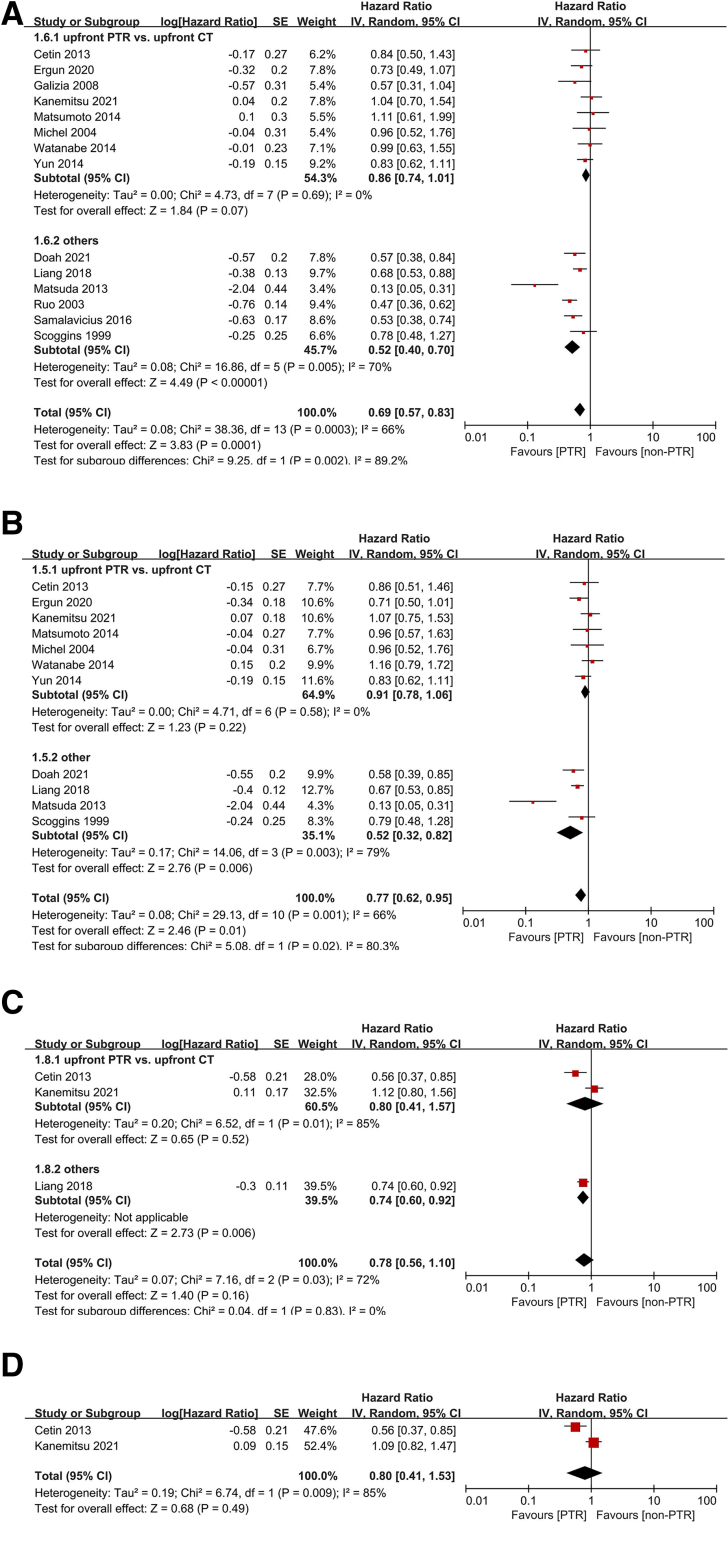
Forest plots for 3-year OS (**A**), 5-year OS (**B**), 2-year PFS (**C**), and 3-year PFS (**D**).

Of all included studies, six non-RCTs reported a significant advantage of OS in the PTR group. Compared with non-PTR group, upfront PTR group was associated with better 3-year (HR: 0.69, 95% CI, 0.57–0.83, *P* = 0.0001) (Figure 2A) and 5-year OS (HR: 0.77, 95% CI, 0.62–0.95, *P* = 0.01) (Figure 2B). A high level of heterogeneity was observed in both analyses (*I*^2^ = 66% for both 3-year and 5-year OS).

Subgroup analyses indicated that there was no significant difference between upfront PTR and upfront CT group (*P* = 0.07 and 0.22 for 3-year and 5-year OS), and no heterogeneity was observed.

**Figure 3:**
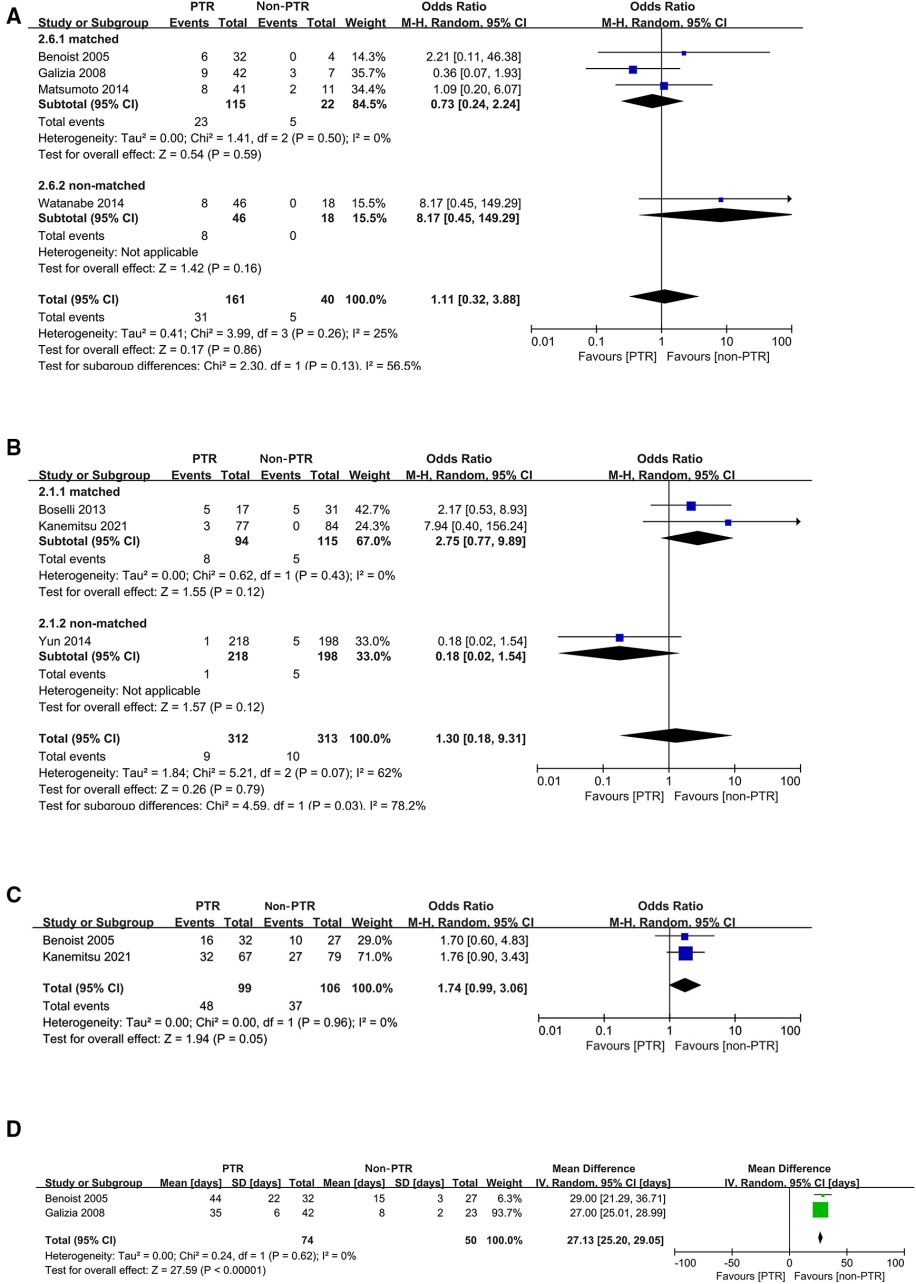
Forest plots for postoperative complications (**A**), 30-day mortality after upfront treatment (**B**), grade 3 or higher adverse effects due to chemotherapy (**C**), and interval from diagnosis to chemotherapy (**D**).

Only three studies reported PFS outcomes between the two groups. There was no significant difference in either 2-year (HR: 0.78, 95% CI, 0.56–1.10, *P* = 0.16) (Figure 2C) or 3-year DFS (HR: 0.80, 95% CI, 0.41–1.53, *P* = 0.49) (Figure 2D). The heterogeneities might be also due to various types of treatment in non-PTR groups.

In addition, four studies (10, 19, 21, 22) reporting a comparison of 2-year OS between groups suggested that PTR was not significantly related to the benefit from survival. Patients in non-PTR groups of all these four studies received CT. One study (32) suggested that 2-year, 3-year, and 5-year OS all benefit from PTR, but did not provide more adequate data for a meta-analysis.

Notably, Zhang et,al (29). reported that PTR could prolonged OS in patients only with left-side CRC (*P* = 0.009) but showed no benefit in terms of right-side CRC (*P* = 0.910).

### Secondary outcomes

In pooled analyses, comparable incidence of postoperative complications (*P* = 0.86) (Figure 3A) and 30-day mortality after upfront treatment (*P* = 0.79) (Figure 3B) were observed. Grade 3 or higher adverse effects due to CT were more frequent in the PTR group with marginal significance (OR: 1.74, 95% CI, 0.99–3.06, *P* = 0.05) (Figure 3C). Additionally, pooled analysis of two studies indicated that patients in non-PTR groups received CT earlier than those in PTR groups (WMD: 27.13 days, 95% CI, 25.20–29.05, *P* < 0.00001) (Figure 3D), and another study (26) similarly reported that the median time from the first visit to chemotherapy was significantly earlier in CT group.

Several studies reported symptoms of the primary tumor over the period of palliative treatment in non-PTR groups, mainly marked by hemorrhage, perforation, and obstruction. The proportions of patients above requiring emergency surgery due to these symptoms ranged from 3% to 27%.

## Discussion

Survival outcomes or the follow-up morbidities may vary considerably because of different treatment manners for asymptomatic patients suffering from unresectable stage IV CRC. Therefore, the strategy of upfront treatment is of great importance for newly diagnosed patients. Even though a significant benefit from PTR was showed in OS, subgroup analysis indicated that there was no significant difference between upfront PTR and upfront CT group. It is also an intriguing finding because a proportion of the patients in non-PTR groups were not treated with CT and some even did not receive any treatment. Besides, the follow-up status of patients without upfront resection would not be inferior to that of patients receiving upfront PTR. Even if patients treated conservatively in the initial diagnosis eventually need surgery due to symptoms of the primary tumor, the postoperative outcomes were relatively acceptable. The only issue that warranted consideration may be the significantly different start times of CT. Granted, the current guideline (34) recommends the startup of CT should be as soon as possible, but a meta-analysis (35) including 3 RCTs published in 2018 assessing survival differences with immediate vs. delayed CT for patients with asymptomatic, unresectable, and metastatic CRC suggested that giving immediate CT might make little or no difference to OS (*P* = 0.18). However, the researchers found it hard to draw a credible conclusion due to the limited number of trials, very sparse data, and uncertainty of the high-level evidence provided. Thus, this issue is still necessary for cautious consideration particularly in the evaluation process of surgical indications.

Considering the heterogeneities observed in meta-analyses of the survival outcomes, we divided the studies that all participants in the non-PTR group receiving CT into separate subgroups and performed sensitivity analyses of the results, consequently yielding a contrary result in OS outcome. Tests for heterogeneity showed no heterogeneity in the CT subgroups in terms of survival analyses, indicating that the results in CT subgroups were more convincing. As for the incidence of postoperative complications and 30-day mortality after upfront treatment, sensitivity analyses were performed that heterogeneity was eliminated by excluding studies whose clinicopathological characteristics were not matched. But in any case, the results still showed comparable between the two groups.

Some of the included studies also yielded interesting findings through further analyses. There were five studies (17, 18, 21, 28, 32) reporting significant differences of primary tumor sites between PTR and non-PTR groups. Patients with right-sided colon cancer in four of these studies (17, 18, 28, 32) were more likely to receive PTR, and they all achieved better OS except for studies reported by Michel et al. On the other hand, patients with rectal cancer in another study (21) tended to receive PTR, and the results did not support the significant advantage of PTR. A study (28) reported that the site of the tumor in the rectum was statistically significant independent prognostic factors for better survival. Besides, Liang (30) and his colleges found that wild-type RAS gene was a favorable factor for patients experiencing PTR with asymptomatic unresectable stage IV CRCs by multivariate regression analysis (*P* = 0.039), while the survival outcomes were comparable between the PTR group and non-PTR group for patients with mutant-type RAS (*P* = 0.102). Similarly, BRAF mutation, which is also an essential prognostic marker in stage IV CRC, should be assessed in this type of patient. Additionally, when patients were classified according to the volume of liver parenchyma replaced by metastases, the level of unresectable liver tumor load was not found to favor primary tumor resection (16). As expected by the researchers, the survival outcome was correlated with the size of the liver tumor load. These findings demonstrated that resection of asymptomatic primary lesions did not provide a survival advantage in incurable stage IV disease. However, it is a great pity that no more studies focused on this issue from then on.

Two included studies (19, 20) reported interval between diagnosis and the start of systemic CT in the CT group and period between PTR and the start of systemic CT in the PTR group. Both indicated that PTR would significantly lead to CT delay. One of them (19) reported that 18.9% of patients resulted in postoperative complication after PTR including wound infection, pleural effusion, pulmonary embolism, urinary tract infection, and intra-abdominal abscess. The other (20) showed that 21.4% of patients with PTR experienced slight postoperative complications, but all could be discharged from hospital and were able to undergo CT as scheduled. According to our subgroup analyses, PTR prior to the initiation of systemic CT was not necessarily required. Instead, PTR could bring complications which might delay the application of CT. Furthermore, wasted finances of unnecessary surgery should also be considered as a significant issue from the perspective of health economics.

With the publication of RCTs, an updated systematic review and meta-analysis was urgently required. In recent years, original articles concerning the role of upfront PTR in asymptomatic and unresectable stage IV CRC emerged with conflicting results. Due to the retrospective nature of these reports with discrepancies of clinicopathological features between groups and the lack of randomization applied, significant heterogeneities were observed in previous meta-analyses. Notably, some of these relevant meta-analyses mixed asymptomatic cases with symptomatic or unclear ones. Moreover, we further compared the differences between upfront PTR and upfront CT groups and found that upfront PTR was not necessary for patients who were willing to receive CT. This finding meets the conclusions of both RCTs.

The selection of target participants for further studies may need to be more precise. For example, the inclusion of patients with no or slight symptoms is demanded. Lam-Boer and his colleges suggested an OS benefit for patients with unresectable stage IV CRCs who underwent PTR as the upfront treatment after diagnosis in a nationwide population-based propensity-score adjusted study, but they also analyzed that confounding by indication could not be avoided as in most observational studies. Without available data on clinical symptoms, it could be assumed that patients with symptomatic CRCs were more likely to undergo PTR. Besides, with the innovation of anti-cancer drugs, a variety of alternatives can thus opt for conservative treatment without PTR. To determine which treatment approach is the key to providing better survival outcomes, subgroup analyses involving specific treatment regimens should be explored.

There were serval limitations in this study. Firstly, the majority of the included studies were retrospective studies leading to the lack of randomization and control of baseline data. Secondly, both RCTs drawing conclusions that PTR showed no survival benefit were based on East Asian populations, which has the potential for racial bias. Thirdly, radiofrequency ablation for liver metastasis (36) and radiotherapy for rectal cancer (37) may play a certain role for unresectable metastatic CRC patients. Although these treatments were partially addressed in the included studies, they were not analyzed independently, making it difficult to clarify their effects. Lastly, significant heterogeneities were shown, which might be blamed on differences in study design, sample sizes, treatment approaches in non-groups, and clinicopathological features including the primary site of tumor and severity of tumor metastases.

In any case, the role of PTR in outcomes improvement will remain an area of ongoing debate for years. It is worth noting that there are still ongoing trials such as the CAIRO4 study (38), but more prospective and randomized studies are urgently needed for better assessment.

## Conclusions

PTR might be related to improved OS for asymptomatic patients with unresectable stage IV CRC, whereas receiving upfront CT is a rational alternative without detrimental influence on survival or adverse outcomes compared with upfront PTR.

## Data Availability

The original contributions presented in the study are included in the article/**Supplementary Material**, further inquiries can be directed to the corresponding author/s.
