# Supplementary material for: The role of upfront primary tumor resection in asymptomatic patients with unresectable stage IV colorectal cancer: A systematic review and meta-analysis
Source: Front Surg. 2023 Jan 6;9:1047373. doi: 10.3389/fsurg.2022.1047373 (PMC9857770; doi:10.3389/fsurg.2022.1047373)
Supplement: Supplementary file 1 [file Datasheet1.pdf]

Supplementary Table 1: Database searched from Pubmed (1990 - August 2021)

#	Searches
1	"Colorectal Neoplasms"[Mesh]
2	((((((((((((((Colorectal Neoplasm[Title/Abstract]) OR (Neoplasm, Colorectal[Title/Abstract])) OR (Neoplasms, Colorectal[Title/Abstract])) OR (Colorectal Tumors[Title/Abstract])) OR (Colorectal Tumor[Title/Abstract])) OR (Tumor, Colorectal[Title/Abstract])) OR (Tumors, Colorectal[Title/Abstract])) OR (Colorectal Cancer[Title/Abstract])) OR (Cancer, Colorectal[Title/Abstract])) OR (Cancers, Colorectal[Title/Abstract])) OR (Colorectal Cancers[Title/Abstract])) OR (Colorectal Carcinoma[Title/Abstract])) OR (Carcinoma, Colorectal[Title/Abstract])) OR (Carcinomas, Colorectal[Title/Abstract])) OR (Colorectal Carcinomas[Title/Abstract])
3	1 OR 2
4	"Neoplasm Metastasis"[Mesh]
5	((((((((Neoplasm Metastases[Title/Abstract]) OR (Metastases, Neoplasm[Title/Abstract])) OR (Metastasis, Neoplasm[Title/Abstract])) OR (Metastase[Title/Abstract])) OR (Metastases[Title/Abstract])) OR (Metastasis[Title/Abstract])) OR (Stage IV[Title/Abstract])) OR (stage 4[Title/Abstract])) OR (stage four[Title/Abstract])
6	4 OR 5
7	(((((palliat*[Title/Abstract]) OR (irresect*[Title/Abstract])) OR (unresect*[Title/Abstract])) OR (nonresect*[Title/Abstract])) OR (non-resect*[Title/Abstract])) OR (incurab*[Title/Abstract])
8	(asympt*[Title/Abstract]) OR (sympt*[Title/Abstract])
9	3 AND 6 AND 7 AND 8
**Total hits**	**539**

Supplementary Table 2: Database searched from Embase (1990 - August 2021)

#	**Searches**
1	'colorectal tumor'/exp
2	'colorectal neoplasm':ti,ab OR 'neoplasm, colorectal':ti,ab OR 'neoplasms, colorectal':ti,ab OR 'colorectal tumors':ti,ab OR 'colorectal tumor':ti,ab OR 'tumor, colorectal':ti,ab OR 'tumors, colorectal':ti,ab OR 'colorectal cancer':ti,ab OR 'cancer, colorectal':ti,ab OR 'cancers, colorectal':ti,ab OR 'colorectal cancers':ti,ab OR 'colorectal carcinoma':ti,ab OR 'carcinoma, colorectal':ti,ab OR 'carcinomas, colorectal':ti,ab OR 'colorectal carcinomas':ti,ab
3	1 OR 2
4	'metastasis'/exp
5	'metastasis':ti,ab OR 'metastases':ti,ab OR 'metastase':ti,ab OR 'stage iv':ti,ab OR 'stage four':ti,ab OR 'stage 4':ti,ab
6	4 OR 5
7	'palliat*':ti,ab OR 'irresect*':ti,ab OR 'unresect*':ti,ab OR 'nonresect*':ti,ab OR 'non-resect':ti,ab OR 'incurab*':ti,ab
8	'sympt*':ti,ab OR 'asympt*':ti,ab
9	3 AND 6 AND 7 AND 8
**Total hits**	**665**

Supplementary Table 3: Database searched from Cochrane Library (1990 - August 2021)

#	**Searches**
1	MeSH descriptor: [Colorectal Neoplasms] explode all trees
2	Colorectal Neoplasm:ti,ab,kw OR Neoplasm, Colorectal:ti,ab,kw OR Neoplasms, Colorectal:ti,ab,kw OR Colorectal Tumors:ti,ab,kw OR Colorectal Tumor:ti,ab,kw OR Tumor, Colorectal:ti,ab,kw OR Tumors, Colorectal:ti,ab,kw OR Colorectal Cancer:ti,ab,kw OR Cancer, Colorectal:ti,ab,kw OR Cancers, Colorectal:ti,ab,kw OR Colorectal Cancers:ti,ab,kw OR Colorectal Carcinoma:ti,ab,kw OR Carcinoma, Colorectal:ti,ab,kw OR Carcinomas, Colorectal:ti,ab,kw OR Colorectal Carcinomas:ti,ab,kw
3	1 OR 2
4	MeSH descriptor: [Neoplasm Metastasis] explode all trees
5	Neoplasm Metastases:ti,ab,kw OR Metastases, Neoplasm:ti,ab,kw OR Metastasis, Neoplasm:ti,ab,kw OR Metastase:ti,ab,kw OR Metastases:ti,ab,kw OR Metastasis:ti,ab,kw OR Stage IV:ti,ab,kw OR Stage 4:ti,ab,kw OR Stage four:ti,ab,kw
6	4 OR 5
7	"palliat*":ti,ab,kw OR "irresect*":ti,ab,kw OR "unresect*":ti,ab,kw OR "nonresect*":ti,ab,kw OR "non-resect":ti,ab,kw OR "incurab*":ti,ab,kw
8	"sympt*":ti,ab,kw OR “asympt*”:ti,ab,kw
9	3 AND 6 AND 7 AND 8
**Total hits**	**90**

Supplementary Table 4: Database searched from Web of Science (1990 - August 2021)

#	**Searaches**
1	TS=(Colorectal Neoplasm* OR neoplasm colorectal OR neoplasms colorectal OR colorectal tumor* OR tumor colorectal OR tumors colorectal OR colorectal cancer OR cancer colorectal OR cancers colorectal OR colorectal cancers OR colorectal carcinoma OR carcinoma colorectal OR carcinomas colorectal OR colorectal carcinomas)
2	TS=(Neoplasm Metastasis OR Neoplasm Metastases OR Metastases, Neoplasm OR Metastasis, Neoplasm OR Metastase* OR Metastasis OR Stage IV OR stage 4 OR stage four)
3	TS=(palliat* OR irresect* OR unresect* OR nonresect* OR non-resect* OR incurab*)
4	TS=(sympt* OR asympt*)
5	3 AND 6 AND 7 AND 8
**Total Hits**	**711**

Supplementary Table 5: Methodological quality and risk of bias

Study	Modified Jadad Score	New Castle Ottawa(NOS)			
		Selection (0-4)	Comparability (0-2)	Outcome (0-3)	Total (0-9)
Scoggins, 1999		***		**	5
Ruo, 2003		***		**	5
Michel, 2004		***		**	5
Benoist, 2005		**	**	*	5
Galizia, 2008		**	**	***	7
Seo, 2010		***	*	***	7
Boselli, 2013		**	**	**	6
Cetin, 2013		**	**	**	6
Matsuda, 2013		**		**	4
Matsumoto, 2014		***	**	***	8
Watanabe, 2014		***		***	6
Yun, 2014		***	**	***	8
Samalavicius, 2016		***		**	5
Zhang, 2017		**		**	4
Liang, 2018		***	*	***	7
Ergun, 2020		*		***	4
Park, 2020	5				
Urvay, 2020		***	*	***	7
Doah, 2021		**	**	***	7
Kanemitsu, 2021	5				

Supplementary Table 6: Full-text articles excluded

Reasons	Number of studies	Reference
Cases included with symptom	12	(1-12)
Cases included without symptomatic or asymptomatic definition	9	(13-21)
Incomplete data	1	(22)
Full text unavailable	1	(23)
Only chemotherapy	1	(24)

References of excluded studies

1. Shida D, Boku N, Tanabe T, Yoshida T, Tsukamoto S, Takashima A, et al. Primary Tumor Resection for Stage IV Colorectal Cancer in the Era of Targeted Chemotherapy. Journal of gastrointestinal surgery : official journal of the Society for Surgery of the Alimentary Tract. 2019;23(11):2144-50.
2. Maroney S, de Paz CC, Reeves ME, Garberoglio C, Raskin E, Senthil M, et al. Benefit of Surgical Resection of the Primary Tumor in Patients Undergoing Chemotherapy for Stage IV Colorectal Cancer with Unresected Metastasis. Journal of gastrointestinal surgery : official journal of the Society for Surgery of the Alimentary Tract. 2018;22(3):460-6.
3. Lau JWL, Chang HSY, Lee KY, Gwee YX, Lee WQ, Chong CS. Survival outcomes following primary tumor resection for patients with incurable metastatic colorectal carcinoma: Experience from a single institution. Journal of Digestive Diseases. 2018;19(9):550-60.
4. t Lam-Boer J, Van der Geest LG, Verhoef C, Elferink ME, Koopman M, de Wilt JH. Palliative resection of the primary tumor is associated with improved overall survival in incurable stage IV colorectal cancer: A nationwide population-based propensity-score adjusted study in the Netherlands. International journal of cancer. 2016;139(9):2082-94.
5. Shida D, Hamaguchi T, Ochiai H, Tsukamoto S, Takashima A, Boku N, et al. Prognostic Impact of Palliative Primary Tumor Resection for Unresectable Stage 4 Colorectal Cancer: Using a Propensity Score Analysis. Annals of surgical oncology. 2016;23(11):3602-8.
6. Kim MS, Chung M, Ahn JB, Kim CW, Cho MS, Shin SJ, et al. Clinical significance of primary tumor resection in colorectal cancer patients with synchronous unresectable metastasis. Journal of surgical oncology. 2014;110(2):214-21.
7. Caceres M, Pascual M, Alonso S, Montagut C, Gallen M, Courtier R, et al. Treatment of colorectal cancer with unresectable metastasis with chemotherapy without primary tumor resection: Analysis of tumor-related complications. Cirugia espanola. 2014;92(1):30-7.
8. Chan TW, Brown C, Ho CC, Gill S. Primary tumor resection in patients presenting with metastatic colorectal cancer: Analysis of a provincial population-based cohort. American Journal of Clinical Oncology: Cancer Clinical Trials. 2010;33(1):52-5.
9. Cellini C, Hunt SR, Fleshman JW, Birnbaum EH, Bierhals AJ, Mutch MG. Stage IV rectal cancer with liver metastases: is there a benefit to resection of the primary tumor? World journal of surgery. 2010;34(5):1102-8.
10. Poultsides GA, Servais EL, Saltz LB, Patil S, Kemeny NE, Guillem JG, et al. Outcome of primary tumor in patients with synchronous stage IV colorectal cancer receiving combination chemotherapy without surgery as initial treatment. Journal of Clinical Oncology. 2009;27(20):3379-

84.
11. Kaufman MS, Radhakrishnan N, Roy R, Gecelter G, Tsang J, Thomas A, et al. Influence of palliative surgical resection on overall survival in patients with advanced colorectal cancer: a retrospective single institutional study. Colorectal disease : the official journal of the Association of Coloproctology of Great Britain and Ireland. 2008;10(5):498-502.
12. Konyalian VR, Rosing DK, Haukoos JS, Dixon MR, Sinow R, Bhaheetharan S, et al. The role of primary tumour resection in patients with stage IV colorectal cancer. Colorectal disease : the official journal of the Association of Coloproctology of Great Britain and Ireland. 2007;9(5):430-7.
13. Li C-L, Tang D-R, Ji J, Zang B, Chen C, Zhao J-Q. Colorectal adenocarcinoma patients with M1a diseases gain more clinical benefits from palliative primary tumor resection than those with M1b diseases: A propensity score matching analysis. World journal of clinical cases. 2020;8(15):3230-U122.
14. Xu Z, Becerra AZ, Fleming FJ, Aquina CT, Dolan JG, Monson JR, et al. Treatments for Stage IV Colon Cancer and Overall Survival. The Journal of surgical research. 2019;242:47-54.
15. van Rooijen KL, Shi Q, Goey KKH, Meyers J, Heinemann V, Diaz-Rubio E, et al. Prognostic value of primary tumour resection in synchronous metastatic colorectal cancer: Individual patient data analysis of first-line randomised trials from the ARCAD database. European journal of cancer (Oxford, England : 1990). 2018;91:99-106.
16. Lau JWL, Chang HSY, Lee KY, Gwee YX, Lee WQ, Chong CS. Modern-day palliative chemotherapy for metastatic colorectal cancer: does colonic resection affect survival? ANZ journal of surgery. 2018;88(11):E772-E7.
17. Gulack BC, Nussbaum DP, Keenan JE, Ganapathi AM, Sun Z, Worni M, et al. Surgical Resection of the Primary Tumor in Stage IV Colorectal Cancer Without Metastasectomy is Associated With Improved Overall Survival Compared With Chemotherapy/Radiation Therapy Alone. Diseases of the colon and rectum. 2016;59(4):299-305.
18. Xu H, Xia Z, Jia X, Chen K, Li D, Dai Y, et al. Primary Tumor Resection Is Associated with Improved Survival in Stage IV Colorectal Cancer: An Instrumental Variable Analysis. Scientific reports. 2015;5:16516.
19. Tarantino I, Warschkow R, Worni M, Cerny T, Ulrich A, Schmied BM, et al. Prognostic Relevance of Palliative Primary Tumor Removal in 37,793 Metastatic Colorectal Cancer Patients A Population-based, Propensity Score-adjusted Trend Analysis. Annals of surgery. 2015;262(1):112-20.
20. Venderbosch S, de Wilt JH, Teerenstra S, Loosveld OJ, van Bochove A, Sinnige HA, et al. Prognostic Value of Resection of Primary Tumor in Patients with Stage IV Colorectal Cancer: Retrospective Analysis of Two Randomized Studies and a Review of the Literature. Annals of surgical oncology. 2011;18(12):3252-60.
21. Ichikawa N, Homma S, Yoshida T, Mitsuhashi T, Iijima H, Ogasawara K, et al. An increase in the peripheral lymphocyte-to-monocyte ratio after primary site resection is associated with a prolonged survival in unresectable colorectal carcinoma. Surgery today. 2020;50(6):604-14.
22. Yun HR, Lee WY, Lee WS, Cho YB, Yun SH, Chun HK. The prognostic factors of stage IV colorectal cancer and assessment of proper treatment according to the patient's status. International journal of colorectal disease. 2007;22(11):1301-10.
23. Jonsson G, Philipson L, Villman K, Valachis A. Upfront Radiotherapy in Patients With Asymptomatic Incurable Rectal Cancer:. Retrospective Cohort Study. Anticancer research.

2020;40(10):5853-60.
24. Muratore A, Zorzi D, Bouzari H, Amisano M, Massucco P, Sperti E, et al. Asymptomatic colorectal cancer with un-resectable liver metastases: immediate colorectal resection or up-front systemic chemotherapy? Annals of surgical oncology. 2007;14(2):766-70.

Supplementary Figure 1: Funnel plot for 3-year OS and 5-year OS

Supplementary Figure 1

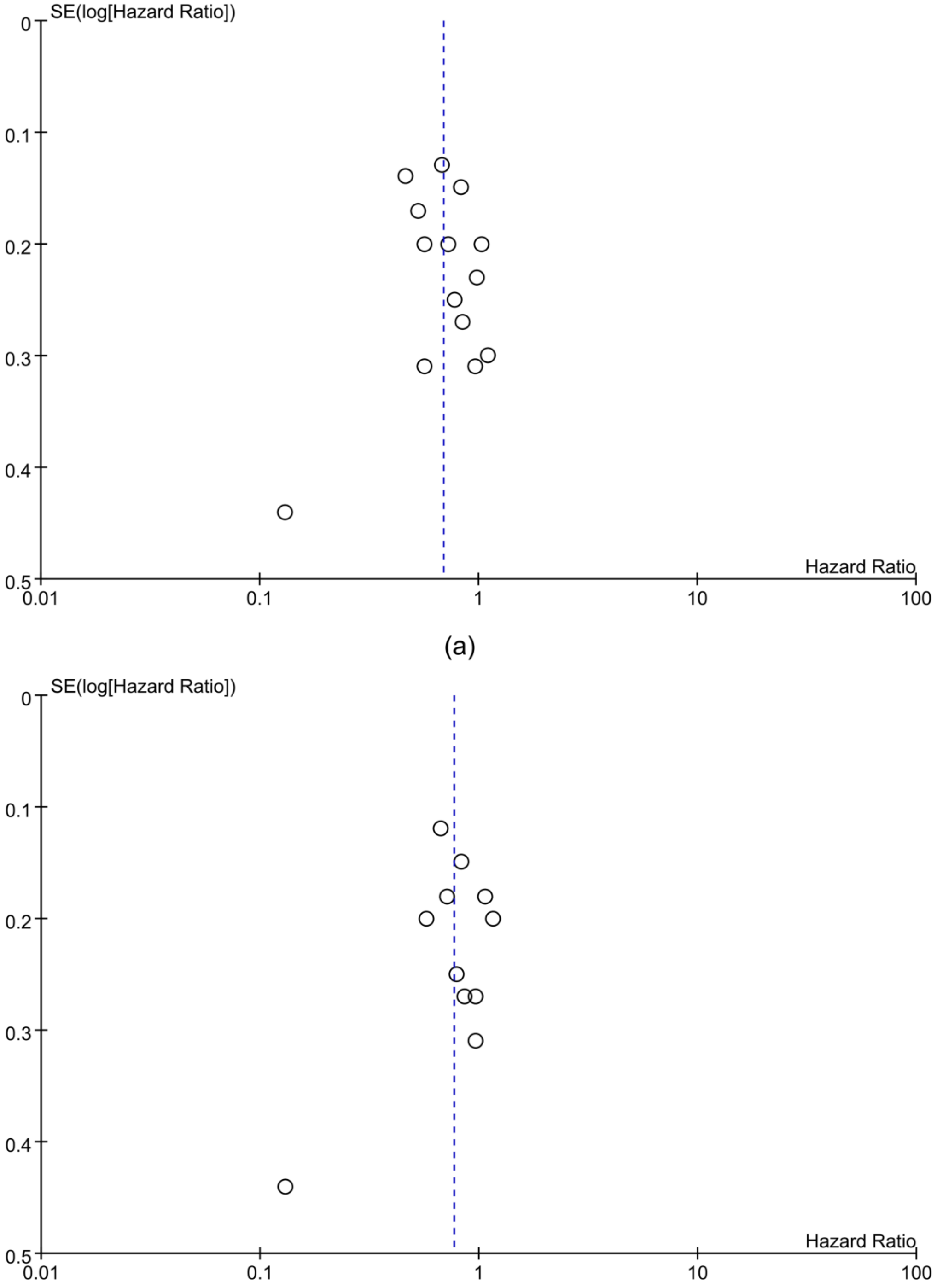

(b)

Funnel plots for 3-year OS (a) and 5-year OS (b)
